# Determination of Alanine Aminotransferase with an Electrochemical Nano Ir-C Biosensor for the Screening of Liver Diseases

**DOI:** 10.3390/bios1030107

**Published:** 2011-07-12

**Authors:** Chang-Jung Hsueh, Joanne H. Wang, Liming Dai, Chung-Chiun Liu

**Affiliations:** 1 Department of Chemical Engineering and Electronics Design Center, Case Western Reserve University, 10900 Euclid Avenue, Cleveland, OH 44106, USA; E-Mails: cxh300@case.edu (C.-J.H.); liming.dai@case.edu (L.D.); 2 Department of Biology, Brown University, 69 Brown Street, Providence, RI 02912, USA; E-Mail: joanne_wang@brown.edu (J.H.W.)

**Keywords:** liver diseases, alanine aminotransferase, electrochemical detection, biosensor

## Abstract

Alanine aminotransaminase (ALT), is an enzyme that normally resides in serum and body tissues, especially in the liver. It is released into the serum as a result of tissue injury; hence the concentration of ALT in the serum may be increased with acute damage to hepatic cells. A single use, disposable biosensor, comprising iridium nano-particle as catalyst dispersed on carbon paste, has been developed for the determination of ALT concentration. The biosensor is based on quantifying H_2_O_2_ concentration produced by a serial of ALT enzymatic reactions. It operates well at room temperature in different physiological fluids: phosphate buffer, calf serum and human serum for ALT concentration of 0–544 ng/mL. Experimental results in human serum are compared to those obtained by spectrophotometric assays with excellent agreement. Therefore, the Ir/C biosensor shows good relationship on the dilution of concentrated ALT clinical applications.

## 1. Introduction

Alainine aminotransferase (ALT) is an enzyme which catalyzes the conversion of alanine and α-ketoglutarate to pyruvate and glutamate contributing to cellular nitrogen metabolism and liver gluconeogenesis. ALT releases into the blood stream, elevating the levels of ALT to abnormally high concentration [1]. Therefore, the measurement of ALT levels in human serum has proved to be a valuable indicator of liver function in clinical settings. Normal level of ALT in the bloodstream typically range from 5–35 UL^−1^, following liver damage the enzyme can be up to 50 fold the normal range (250–1,400 UL^−1^) [2].

**Scheme 1:**
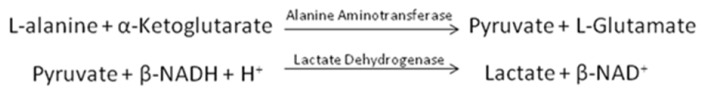
Reaction mechanism for the quantization of Alainine aminotransferase (ALT) spectrophotometrically.

The current clinical method of quantifying ALT is the spectrophotometric method requiring the reaction [3] shown in Scheme 1.

Spectrophotometric detection is the widely adopted clinical standard method in the determining the serum concentration of ALT. In this detection method, the measurement of the absorbance change of NADH concentration at 340 nm UV light is used based on the pyruvate reaction with lactate dehydrogenase (LDH). However, spectrophotometric methods require physically large and expensive instruments, skillful technician, as well as relatively large sample volume around a few milliliters. Thus, the spectrophotometric analysis is not suitable for point of care or home-use detection of ALT.

Ideally, ALT detection can be accomplished with a relatively small-scale, portable, inexpensive, and disposable sensor that utilizes a simple detection method. Jamal *et al*. demonstrated an electrochemical biosensor using a three-step reaction mechanism which requires the re-oxidation of the mediator, ferrocene carboxylic acid, as the source of current response [4]. This method may provide sensitive detection of ALT in the range of 25–150 UL^−1^, and the reaction mechanism is complicated. Other electrochemical detection methods use a simpler two-step reaction mechanisms, such as the “glutamate sensors” proposed by Song *et al*., which apply glutamate oxidase (GluOx) to produce H_2_O_2_ from the L-glutamate produced by ALT , and quantifications of ALT can be further measured by the oxidation or reduction of H_2_O_2_ [5,6]. In the method proposed by Chang *et al*., palladium is material selected as the working electrode [7], and a Nafion membrane is applied to further prevent ascorbic acid from reaching the electrode [8,9]. The increase in the quantity of the Nafion decreases the sensor response.

**Scheme 2:**
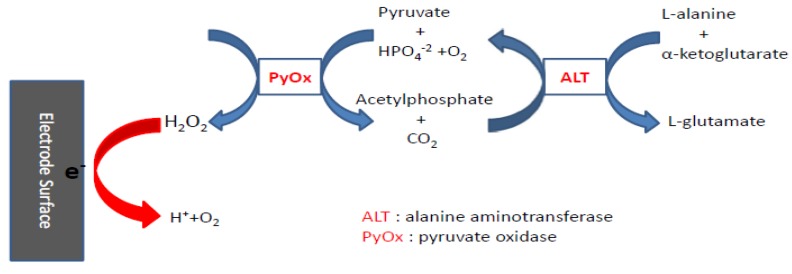
Reaction mechanism to detect ALT electrochemically requires the action of pyruvate oxidase to generate H_2_O_2_ from pyruvate, the product of the reaction catalyzedby ALT.

An alternative method to the mechanism proposed by Song *et al.* [5] is the two-step reaction mechanism shown in Scheme 2.

Similar to the reaction mechanism of glutamate sensor described, the production of pyruvate driven by ALT can be converted to hydrogen peroxide under the catalytic reaction of pyruvate oxidase (PyOx), which can be further electrochemically oxidized to quantify the concentration of ALT. This approach has been used by Xuan *et al*. [10]. This detection method provides a wider range at lower concentrations of ALT, and the construction of anti-ALT antibody membrane is not only rather complicated, but also there are some challenges in the layer-by-layer film technique [11].

Metallic nano-particles (NPs) supported by active carbon can be an excellent catalyst for reactions including enzymatically-produced species such as H_2_O_2_ and NADH (nicotinamide adenine dinucleotide, reduced form). Incorporation of unique characteristics of NPs into biosensor can enhance both sensitivity and selectivity [12]. Over the years, we have developed a single use, disposable and screen printed biosensor platform technology [13,14,15,16]. In this development, a 2–5% by weight of iridium (actually iridium oxide) nano-catalyst is added to the active carbon forming the screen printable ink. This biosensor prototype can be used to detect enzymatically-produced H_2_O_2_ at a relatively low oxidation potential minimizing the potential interference by other species. The fabrication of this biosensor prototype has been described elsewhere [13,14,15,16]. 

Our objective in this research is to develop a detection method for ALT using the simple two-step reaction mechanism as shown in Scheme 2 in combination of a single use (eliminating the electrode interference problem due to repeated uses), disposable, cost effective screen-printing biosensor for ALT measurement. This biosensor will be useful for clinical or point-of-care quantification of ALT in human serum. The experimental approach and results are given. Furthermore, the experimental quantifications of ALT in human serum of this biosensor prototype are compared to the “gold standard” spectrophotometric assays of a clinical laboratory, and the results are in excellent agreement. Thus, this single use, disposable, biosensor provides a simple detection method for quantification of ALT in human serum as an excellent technology for clinical and point of care ALT detection.

## 2. Experimental Section

### 2.1. Materials and Reagents

L-Alanine, α-ketoglutararic acid disodium salt, sodium pyruvate, magnesium chloride, flavin adenine dinucleotide [FAD], thiamine phosphate [TPP], pyruvate oxidase [PyOx] (E.C. 1.2.3.3), alanine aminotransferase [ALT] (E.C. 2.6.1.2) and human serum were purchased from Sigma-Aldrich (St. Louis, MO, USA). Bovine calf serum was purchased from Invitrogen (San Diego, CA, USA). Potassium chloride, 3% hydrogen peroxide solution, sodium phosphate monobasic, and sodium phosphate dibasic heptahydrate were purchased from Fisher Scientific (Hampton, NH). The Ir/C particles (5% Ir) were purchased from BASF (Somerset, NJ). The additional chemicals used were of analytical grade. The buffers and solutions made were prepared using deionized water. 

### 2.2. Thick-Film Screen-Printed Prototype

**Figure 1:**
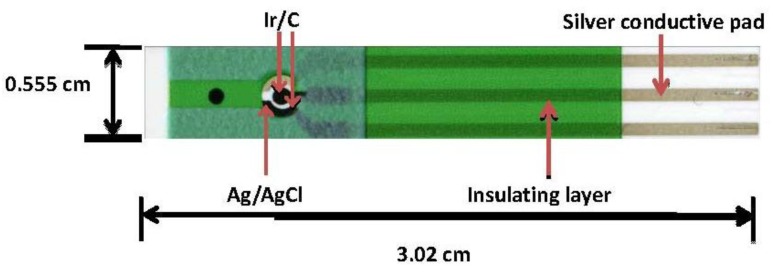
Sensor prototype used for the ALT detection [13,14,15,16].

The formulation of the Ir/C ink used for the working and counter electrodes has been discussed in previous publications [13,14,15,16]. As described, a 787 mm × 584 mm polyester sheet was for the substrate of the sensor on which surface printed silver ink was used as the electrical contact. The biosensor prototype had an overall dimension of 30 mm × 5.5 mm encompassing three electrodes: a Ag/AgCl printed reference electrode, a printed Ir/C working and a Ir/C counter electrodes. The geometric surface area for the printed working electrode was 7.85 × 10^−3^ cm^2^. Figure 1 shows the sensor prototype and will be used for ALT detection in this study.

### 2.3. Experimental Testing Procedure

A CH Instrument 660C workstation (CH Instrument, Inc., Austin, TX) was used for the cyclic voltammetric and amperometric evaluations. All tests were performed at ambient temperature (approximately 21–23 °C). A 200 µL sample volume of different ALT solution was placed in a 2.0 mL microcentrifuge tube. A testing solution containing 250 mM L-alanine, 2.5 mM α-ketoglutarate and 1,760 UL^−1^ of PyOx, in addition to the ALT solution of variable concentration 0–544 ng/mL, which corresponds to ALT specific activity 0–80 UL^−1^. The sensor was inserted into the testing tube to quantify the ALT level. A new fresh sensor was used for each measurement. Each concentration was tested at least three times for the reproducibility. Potential values reported throughout this study were measured *versus* the screen-printed Ag/AgCl reference electrode on the sensor prototype.

## 3. Results and Discussion

### 3.1. H_2_O_2_ Detection in Buffer Solution

The ALT concentration in the solution is proportional to the enzymatically-produced H_2_O_2_. In our previously study, this IrO/C sensor prototype works very well in the detection of H_2_O_2_. In this study, cyclic voltammetry was used to evaluate H_2_O_2_ detection in a scan rate 10 mV/s within the voltage window of −0.1 to +0.4 V *versus* the Ag/AgCl reference electrode. 

**Figure 2:**
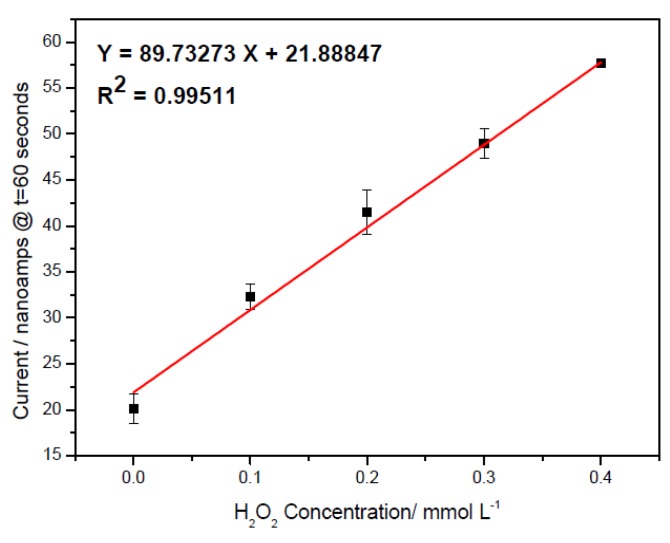
Calibration plot obtained at 60 s for the H_2_O_2_ measurement in the 0.1 M pH 7.5 phosphate buffer with 150 mM KCl supporting electrolyte and reproducibility of the measurement with a new sensor for each measurement n = 3.

Amperometric tests were carried out with 0.1 M pH 7.5 phosphate buffer solutions with a 150 mM KCl as a supporting electrolyte at the H_2_O_2_ concentrations from 0 to 0.4 mmol L^−1^. An operation time of 60 s, at which the minimum steady-state response current was reached, was chosen to record the oxidation current of the H_2_O_2_. The reproducibility of the measurements was evaluated using a new biosensor prototype and each H_2_O_2_ concentration for 3 to 5 times. Figure 2 shows the experimental results, and the linear relationship between the current outputs of the biosensor prototype and the H_2_O_2 _concentrations exists. 

### 3.2. Determination of Pyruvate Concentration with Enzymatically Generated H_2_O_2_

**Figure 3:**
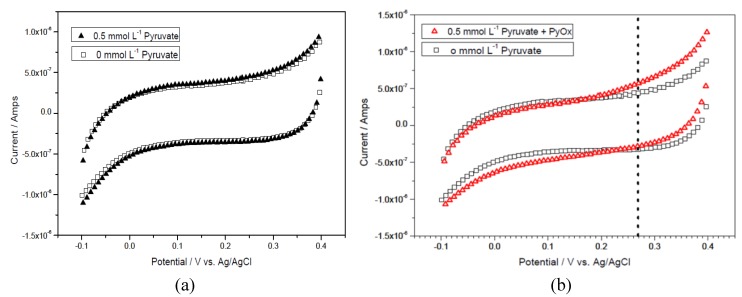
(**a**) Cyclic voltammograms of background (0 mmol L^−1^ pyruvate) and 0.5 mmol L^−1^ pyruvate in a basic testing solution. (**b**) Cyclic voltammograms of background (0 mmol L^−1^ pyruvate) and 0.5 mmol L^−1^ pyruvate in a testing solution with 1,760 UL^−1^ PyOx.

Pyruvate is one of the products from the first reaction and a reactant in the second reaction in the reaction mechanism shown in Scheme 2. Pyruvate will then react with PyOx producing H_2_O_2_ which can then be quantified electrochemically. Therefore, the quantification of pyruvate is necessary in the detection of ALT based on the reaction mechanism shown in Scheme 2. Typically, a new biosensor prototype (Figure 1) was placed inside a 2.0 mL centrifugal tube containing a 0.1 M pH 7.5 phosphate buffer solution with 150 mM KCl supporting electrolyte as well as 1 mM MgCl_2_, 0.2 mM TPP, and 15 µM FAD (defined as **basic testing solution** which contains all the cofactors in the pyruvate reaction). Cyclic voltammetry was scanned from −0.1 to +0.4 at a scan rate of 10 mV/s to determine the potential at which pyruvate would be oxidized enzymatically. Figure 3(a) shows the cyclic voltammograms of the basic testing solution without and with 0.5 mmol L^−1^ pyruvate. Figure 3(a) shows that the cofactors and pyruvate do not contribute to any current measured by the biosensor prototype. Figure 3(b) shows the cyclic voltammograms of the basic testing solution and 0.5 mmol L^−1^ pyruvate in the absence and presence of 1,760 UL^−1^ of enzyme, PyOx. As shown in Figure 3(b) at +0.27 volt *versus* the printed Ag/AgCl reference electrode, the enzymatically-produced H_2_O_2_ was oxidized and the oxidation current could be used to quantify the pyruvate concentration

**Figure 4:**
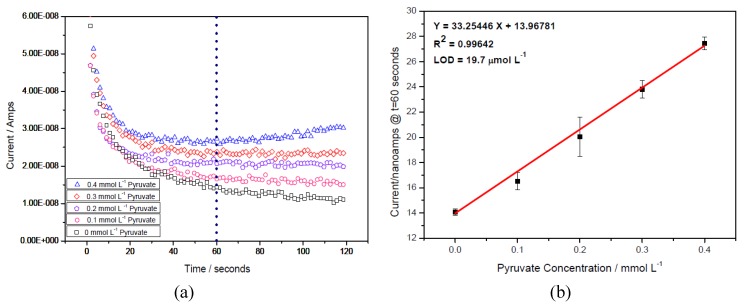
(**a**) Amperometric curves of pyruvate with different concentrations obtained at +0.27 V *versus* Ag/AgCl in a testing solution with 1,760 UL^−1^ PyOx. (**b**) Calibration plot obtained at 60 s for the pyruvate assessment in a testing solution with UL^−1^ PyOx and repeatability of measurement with a new sensor for each measurement n = 3.

These amperometric studies for the quantification of pyruvate concentration were then carried out. Figure 4(a) shows the amperometric measurements of pyruvate at different concentrations in the basic testing solution. Figure 4(b) shows the linear relationship between the current outputs and the pyruvate concentrations indicating that this biosensor can quantify pyruvate effectively and accurately by detecting the enzymatically-produced H_2_O_2_ by PyOx with limit of detection (LOD) = 19.7 µmol L^−1^. 

The selection of time was chosen at the 60th second and then the corresponding current at each pyruvate concentration was plotted as Figure 4(b).

### 3.3. Determination of Alanine Aminotransferase (ALT)

#### 3.3.1. ALT Detection with Cyclic Voltammetry

**Figure 5:**
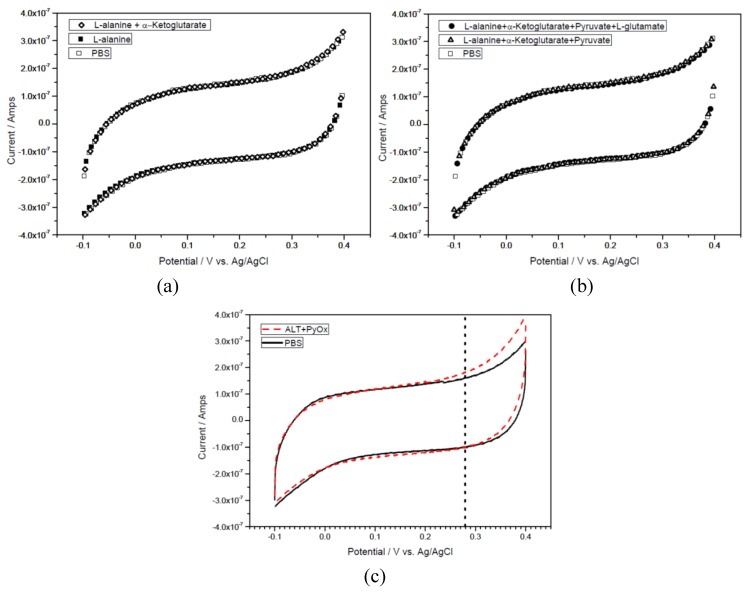
(**a**) Cyclic-voltammetric scans in a testing solution in absence/presence of reactants: L-alanine and α-ketoglutarate. (**b**) Cyclic-voltammetric scans in a testing solution containing different adding species: reactants and products. (**c**) Cyclic-voltammetric scans in a testing solution containing different adding species: reactants in presence of 544 ng/mL ALT and 1,760 UL^−1^ PyOx.

There are chemicals used in the reaction mechanism shown in Scheme 2. Specifically, L-alanine and α-ketoglutarate are the reactants, pyruvate and L-glutamate as the products in the first reaction, phosphate as the co-reactant with pyruvate and acetylphosphate as the products in the second reaction. These elements will not contribute to any current output of the biosensor prototype. Figure 5(a,b) show that there is no contribution of the current output by any of these chemicals. Thus, the current output of the biosensor is exclusively obtained from the oxidation of H_2_O_2_ as shown in Scheme 2. Consequently, the quantity of ALT can be detected based on this reaction mechanism.

Cyclic voltammetric studies of the basic testing solution without or with 1,760 UL^−1^ PyOx and various concentrations of ALT were carried out. It is conclusively determined that these chemicals do not contribute to the enzymatic generated H_2_O_2_ in the reactions shown in Scheme 2. 

#### 3.3.2. ALT Detection with Amperometric Method

In this case of ALT detection, +0.28 V *versus* the printed Ag/AgCl reference electrode was employed based on Figure 5(c) to the amperometric studies in three different fluids: a pH 7.55 phosphate buffer, a 1:1 diluted calf serum and a 1:2 human serum. Both calf serum and human serum were diluted with a pH 7.55 PBS lowering the pH value in solutions. All of these media comprise of 250 mM L-alanine, 2.5 mM α-ketoglutarate and 1,760 UL^−1^ PyOx in the basic testing solution. 

**Figure 6:**
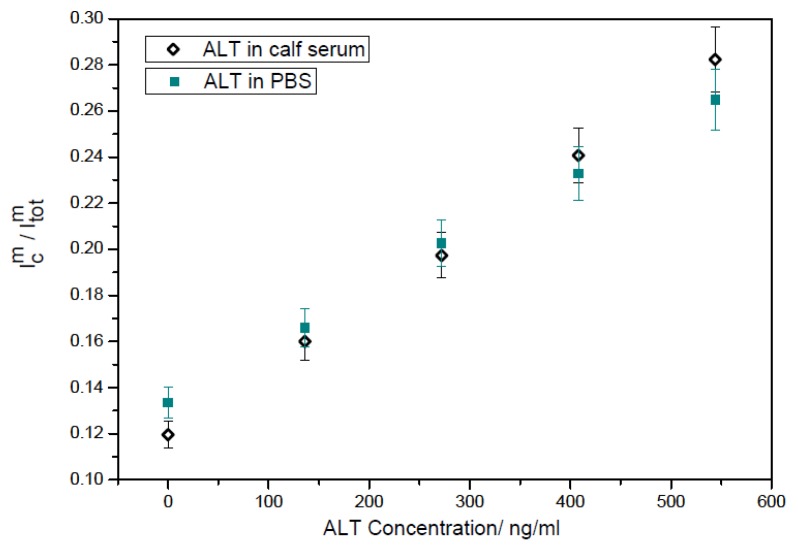
Normalization results for ALT detection in phosphate buffer and calf serum testing.

ALT levels ranging from 0 to 544 ng/mL in each test media was studied. The pH value of the testing solution changed slightly in these media after adding the testing sample resulting at a pH value of 7.35 for the phosphate buffer, a pH 7.4 for the calf serum and a pH 7.65 the human serum. The minute change in pH values of the media would not affect the experimental results significantly. The cyclic voltammetric studies were carried out in the voltage window of −0.1 V to +0.4 V *versus* the printed Ag/AgCl reference electrode with a scan rate of 10 mV per second. The detection of ALT in both PBS and calf serum were consistent with similar corresponding slope with the ALT concentrations. Figure 6 shows a normalized detection linear relationship between the biosensor outputs and the ALT concentrations. The linear relationship shown indicates that this biosensor in combination of the reaction mechanism described in Scheme 2 can be used for a single use, disposable ALT biosensor.

**Figure 7:**
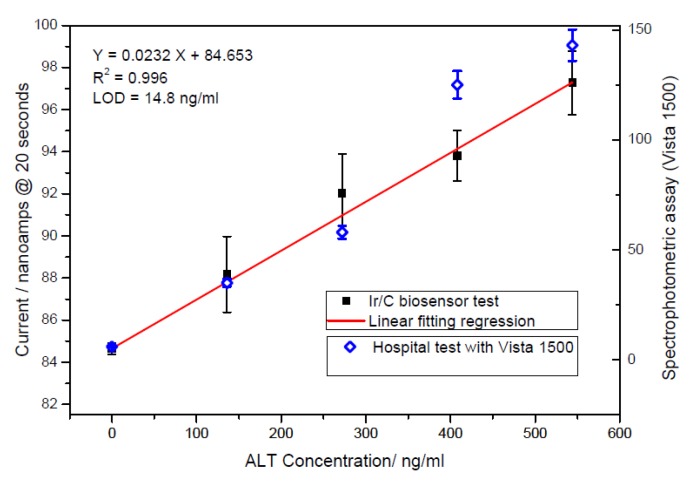
Comparison of measurements obtained by a Vista 1500 spectrophotometer to the Ir/C sensor response currents.

**Figure 8:**
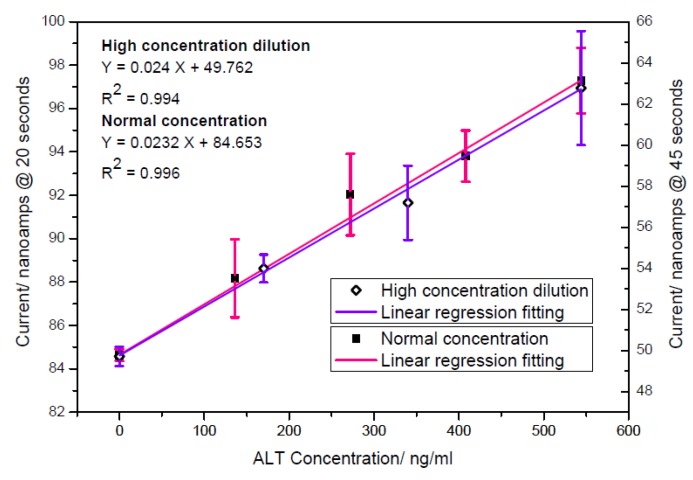
Comparison of measurements made diluting high ALT concentration to the ALT currents response obtained without dilution.

In a realistic clinical application the evaluation of the ALT determination was performed in a human serum by the biosensor and the amperometric currents recorded at 20 s were then compared with the spectrophotometric measurement (Vista 1500) by the University Hospital of Cleveland, Ohio, a “gold standard” for ALT detection in clinical quantification. Figure 7 shows the linear relationship between the obtained response and the ALT concentration in human serum. Additionally, the output of the ALT biosensor was correlated well with the spectrophotometric results, demonstrating that the biosensor prototype can be used effectively in human sample with activity levels over 2-fold upper limits (80 UL^−1^). However, most cases of hepatitis infection and major liver failure could be assessed out several times than normal ALT ranges. Based on practical usage, human serum with ALT concentration levels ranging from 0 to 5.44 µg/mL was subsequently examined. ALT solution was diluted 10-fold smaller than the original ALT added in solution with the same human serum as used for the previous human sample testing. Figure 8 proves that current output of diluting ALT tests are in excellent agreement with the results just showed in Figure 7. Additionally, the limit of detection (LOD = 2.18 UL^−1^) obtained is lower than Jamal’s result (=3.29 UL^−1^) [4]. Consequently, the proposed Ir/C biosensor is suitable for an in vitro ALT quantification in a human sample at a short recording time (less than 1 minute).

## 4. Conclusions

In this study, we have demonstrated the usage of a two-step enzymatic reaction mechanism as shown in Scheme 2 with a highly sensitive Ir/C nano-catalyst single use, disposable, screen-printing biosensor for ALT detection. The detection of ALT was carried out by quantifying the enzymatically-produced H_2_O_2_. The amperometric measurement was at an applied potential of +0.28 V *versus* reference Ag/AgCl electrode at ambient temperature. Experimental results show that the current output of the biosensor and the ALT concentration maintained an excellent linear relationship over the ALT concentration range of 0 to 544 ng/mL. Furthermore, the measurement from the biosensor agreed very well with the clinical “gold standard” spectrophotometric results of ALT in human serum. Also, for the analysis of potentially high ALT concentration, a 10-fold dilution of the sample (human serum) can first be carried out. The biosensor can detect the ALT at lower concentration accurately. Thus this single use, disposable and relatively inexpensive ALT biosensor can be very useful for clinical and point-of-care applications.
